# European Correspondence

**Published:** 1867-11

**Authors:** W. S. Armstrong

**Affiliations:** London


### EUROPEAN CORRESPONDENCE.

LONDON, SEPT. —, 1867.

*Atlanta Medical and Surgical Journal:*

Before leaving Dublin, I had hoped to be able to send you some notes, hastily taken while visiting the medical schools and hospitals there, but my stay was cut shorter than I originally intended in order to remain longer in London. Just now in both these places there is a recess in the schools of medicine—the regular course commencing on the 1st of October.

However, at Dublin I had the pleasuse of meeting Dr. Smith, Prof. of Materia Medica, in the medical department of Trinity College, who was kind enough to fix a day and take me around to examine the College buildings, Library, Museum and Laboratory. With the latter I was much pleased. It is a *very* long room, with a counter in the middle, running the whole length, upon both sides of which are stalls, or little apartments, fitted up with gas, watar and reagents in each, for the use of the class. Upon inquiry, I found that in addition to the lectures usually delivered on chemistry, each student is compelled to devote a certain part of his time—three months of each course—to the study of practical chemistry in the laboratory. For instance, the Professor gives the student a salt for analysis, and he takes it to the little apartment fitted up for him and makes the the analysis. Now it will not take much effort to see the great advantage to be gained by this course of instruction over the one usually pursued. Chemistry is a branch which students are apt to slight and trust to "cramming" and "grinding" (to use a College phrase common among them), just before examination. But when it is taught after this manner, they not only know the benefit of the lectures, but are compelled to have some practical knowledge of science also.

A similar provision has been made for the study of practical Chemistry in one of the schools here, but as yet it is optional with the student to take advantage of it.

The Museum of Materia Medica, almost entirely collected by Prof. Smith, contains specimens of all the drugs and preparations in ordinary use. The principal feature deserving notice in this collection is their classification and arrangement for study. They are put up in glass jars as usual, and so arranged that in one case are all the specimens of animal origin; in the second those of vegetable origin, commencing with the seeds, fruits, flower, bark and root; and in the third are those obtained from the mineral kingdom.

The simplicity and usefulness of such arrangement will be appreciated at a glance. Here the students are required to study and familiarize themselves with the appearance, taste, etc., of the different drugs. Here too, as in chemistry, it is easy to understand the advantage to be gained. For nothing is better understood than the fact that in a practical branch, one demonstration—one thorough ocular examin tion—is worth a dozen descriptions.

In visiting the Hospitals in London, one is struck at a glance, with the frightful number of patients suffering from Phthisis. As Intermittent Fever is *the* affection of a malarial district of country in America, so it seems that Phthisis is *the* disease, at least of that portion of the population here, who are compelled, through poverty, to seek an asylum in the Hospitals during their illness.

One of the symptoms of Typhoid Fever, which Watson dwells on in his work, viz: the *numerous* rose colored spots, I have met with here. It is true, we see them occasionally scattered on the bodies of our patients in America, but here they are to be seen in much greater numbers.

As yet I have met with but little outside of the usual run of cases at this season of the year of especial interest. For the last few days as the weather has grown colder, there has been a perceptible increase in the number of cases of Rheumatism and Pneumonia.

W. S. ARMSTRONG.

